# P-1665. Predictors of Antibiotic Prescription for Upper Respiratory Tract Infections in Cancer Patients

**DOI:** 10.1093/ofid/ofae631.1831

**Published:** 2025-01-29

**Authors:** Hyeon Mu Jang, Aida Boumiza, I Hsin Lin, Nina Cohen, Krishna Shah, Jibran Majeed, Jeffrey Groeger, Susan K Seo

**Affiliations:** University of Ulsan College of Medicine (Asan Medical Center), Seoul, Republic of Korea, Seoul, Seoul-t'ukpyolsi, Republic of Korea; Memorial Sloan Kettering Cancer Center, New York, NY, USA, NYC, New York; Memorial Sloan Kettering Cancer Center, New York, NY, USA, NYC, New York; Memorial Sloan Kettering Cancer Center, New York, NY; Memorial Sloan Kettering Cancer Center, New York, NY, USA, NYC, New York; Memorial Sloan Kettering Cancer Center, New York, NY, USA, NYC, New York; Memorial Sloan Kettering Cancer Center, New York, NY, USA, NYC, New York; Memorial Sloan Kettering, New York, New York

## Abstract

**Background:**

Antibiotic stewardship efforts are ongoing to improve antibiotic prescribing in the outpatient setting for patients with acute upper respiratory infection (URI). Limited data regarding predictors of antibiotic prescription for acute URI are available for ambulatory patients with cancer.

**Methods:**

Adult cancer patients with URI who presented to the Urgent Care Center (UCC) or Symptom Care Clinic (SCC) at Memorial Sloan Kettering Cancer Center and underwent respiratory pathogen panel (RPP) between September 1, 2019, and March 11, 2020, prior to the start of the Coronavirus Disease 2019 pandemic were evaluated. Study population was dichotomized by receipt of antibiotic prescription (yes vs. no). Demographics, cancer treatment, URI characteristics, and URI management were compared by the Student's t test or Mann Whitney Wilcoxon test and Chi-square or Fisher's exact test as appropriate. Logistic regression was performed to evaluate the predictors of antibiotic prescription.

**Results:**

Of 552 patients, 343 (62.1%) had a solid tumor, 384 (69.6%) received cancer treatment within 30 days of the URI, and 377 (68.3%) had a positive RPP result. Antibiotics were prescribed in 156 (28.3%) patients. Only 16.7% (26/156) were appropriate based on the Centers for Disease Control’s Adult Outpatient Treatment Recommendations. Multivariable logistic regression showed that predictors of antibiotic prescribing included SCC visit location (odds ratio (OR) 2.09, 95% confidence interval (CI) 1.30-3.38), symptom duration > 7 days (OR 2.29, 95% CI 1.26-4.17), earache (OR 9.30, 95% CI 3.47-24.93), sinus pain/headache (OR 2.64, 95% CI 1.53-4.56), negative RPP result (OR 1.88, 95% CI 1.18-2.98), and negative RPP result for influenza (OR 3.31, 95% CI 1.53-7.16). Cancer therapy within 30 days of URI diagnosis and pharyngitis were insignificantly associated with antibiotic prescriptions.

**Conclusion:**

Almost one-third of outpatients at a single cancer center were prescribed antibiotics for URI with only 16.7% being appropriate. Getting real-time RPP results can be helpful to optimize antibiotic prescribing. Understanding risk factors for antibiotic prescribing in ambulatory cancer patients with URI may better direct antimicrobial stewardship efforts.

**Disclosures:**

**Susan K. Seo, MD**, Merck: Grant/Research Support

